# The Feasibility of Ambulatory Heart Rate Variability Monitoring in Non‐Suicidal Self‐Injury

**DOI:** 10.1049/htl2.70007

**Published:** 2025-02-28

**Authors:** Alje van Hoorn, Anna Mankee‐Williams, Gareth Lewis, Rafaella Mellili, Jessica Eccles, Cristina Ottaviani, Richard Laugharne, Rohit Shankar

**Affiliations:** ^1^ Department of Intellectual Disability Neuropsychiatry Cornwall Partnership NHS Foundation Trust Truro Cornwall UK; ^2^ Falmouth University Falmouth UK; ^3^ Ospedale Policlinico Universita Bari Apulia Italy; ^4^ Brighton and Sussex Medical School Brighton UK; ^5^ Sussex Partnership NHS Foundation Trust Worthing UK; ^6^ Brighton and Sussex University Hospitals NHS Trust Worthing UK; ^7^ Sapienza University of Rome Rome Italy; ^8^ Cornwall Intellectual Disability Equitable Research (CIDER) University of Plymouth Truro Cornwall UK

**Keywords:** heart rate variability, non‐suicidal self‐injury, self‐harm, vagal tone, patient monitoring, physiological models

## Abstract

The polyvagal theory proposes that the autonomic nervous system influences affective systems and top‐down emotional regulation. Vagal tone, as indexed by heart rate variability (HRV), is a measure of emotion regulation capacity. It is possible that non‐suicidal self‐injury (NSSI) occurs at times of low vagal tone and that NSSI may increase it. Little is known about the feasibility of collecting ambulatory HRV data in the context of NSSI. This prospective observational study examined the feasibility of ambulatory HRV monitoring during NSSI. Ten participants wore a chest‐based heart rate monitor and used a diary app for 1 week. Baseline characteristics were collected. Heart rate monitoring duration, diary app entries, distress scores, and NSSI occurrences were recorded. Participant experience was assessed in a post‐study questionnaire. At baseline, six had a history of NSSI, in two of whom it was current. Ten participants wore the monitor for an average of 137 h. Nine participants successfully used the diary app, making an average of 14 entries over a week. Although no NSSI occurred during the study, the overall experience of participation was positive. It is feasible to monitor HRV and collect app‐based distress scores for a week, including in those with NSSI history.

## Graphical abstract

Vagal tone, as indexed by heart rate variability (HRV), is a measure of emotion regulation capacity. It is possible that non‐suicidal self‐injury (NSSI) occurs at times of low vagal tone and that NSSI may increase it. This prospective observational study examined the feasibility of ambulatory HRV monitoring during NSSI.

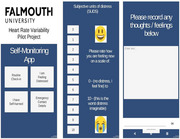

## 1 Introduction

Non‐suicidal self‐injury (NSSI) is defined as repetitive, intentional, direct injury of one's body tissue without suicidal intent that is not socially accepted [1]. It has now been included as a diagnosis in the Diagnostic and Statistical Manual of Mental Disorders (fifth edition) DSM‐5 [2]. It is common with a lifetime prevalence of 17% in a large study of college students [3]. The reasons for NSSI are diverse, but managing distress/affect regulation was found to be a major factor in a meta‐analysis, with more than 90% of studies endorsing this view [4].

### 1.1 Mechanisms Linking NSSI to Affect Regulation

The mechanisms by which NSSI contributes to affect regulation are poorly understood, especially in real‐life situations rather than in a laboratory. This may be due to the difficulties in investigating potentially dangerous behaviour ethically.

Abnormalities in the dopaminergic and opioid systems and a role for addiction pathways have been suggested [5]. An fMRI study of young people showed increased perfusion of brain areas associated with reward and addiction in those who engaged in NSSI compared to controls [6]. There is an association between the hypothalamic pituitary adrenal (HPA) axis and NSSI. In a case report of urinary cortisol in a woman with chronic self‐harm, urinary cortisol levels were found to increase gradually over consecutive nights, culminating in an episode of self‐harm. This was followed by a drop in urinary cortisol, which then increased again until the next episode [7]. None of these proposed mechanisms fully explains why NSSI is associated with affect regulation. Further examination of the phenomenon in vivo could provide valuable mechanistic clues.

### 1.2 The Role of the Autonomic Nervous System

Influential theories propose a role for the autonomic nervous system in emotion regulation. The neurovisceral integration model proposes a link between autonomic, attentional and affective systems in a network involved in emotion regulation. In this model, parasympathetic activity (vagal tone) has a relationship with top‐down emotion regulation of limbic areas by the prefrontal cortex [8].In a study of naval personnel, manipulating HRV with exercise training has been shown to improve prefrontal functioning, which was interpreted in the context of the neurovisceral integration model [9]. It has been shown that increased HRV correlates with prefrontal activation [10]. This relationship may go further than correlated and improved HRV may actually increase prefrontal inhibitory control over subcortical regions [11]. The Polyvagal Theory suggests that the autonomic nervous system provides a substrate for emotional experiences and affective processes [12]. It suggests that vagal nerve activity, via modulation of the social engagement system, regulates emotional state and prosocial behaviour. HRV is an index of cardiac vagal nerve activity (vagal tone) which can be collected non‐invasively [13].

HRV has been proposed as a biomarker of self‐regulation capacity [14]. This is supported by a meta‐analysis of resting HRV in patients with borderline personality disorder (BPD), where emotional instability is common, and HRV was found to be lower than that of controls [15]. The meta‐analysis was based on laboratory studies; it is less clear what happens to HRV in this clinical population in real‐life situations.

Although there are many types of NSSI, including cutting, burning, hitting, scratching and using ligatures, the literature largely treats NSSI as a unitary concept [16]. It is possible that some forms of NSSI, for example, cutting and the use of ligatures, may have vagal effects [17]. Seeing blood is often an important contributor to the relief felt by those who cut themselves [18]. In certain circumstances, the sight of blood is associated with increased vagal tone. In a lab study of the role of perceived control in the face of blood‐injury‐injection stimuli, participants who were less fearful of blood showed greater increases in HRV despite reporting fewer vasovagal symptoms [19].

Ligature use may have vagal effects by means of carotid baroreceptor stimulation. A meta‐analysis of the lifetime prevalence of engagement in self‐asphyxia behaviour (‘choking game’) was found to be 7.4% [20]. Although characterised as a game, links to mental health difficulties are described, including a higher rate of depressive symptoms [21]. A possible clue to the neurobiology of ligature effects comes from a study of carotid baroreceptor stimulation. Individual differences in basal HRV predicted the degree to which right carotid stimulation attenuated amygdala responses during fear processing [22]. Given that carotid sinus massage has vagal effects [23], it would be useful to examine whether the use of ligatures elicited parasympathetic changes.

Further examination of HRV at the time of various forms of NSSI may indicate a role for autonomic mechanisms in emotion regulation in NSSI.

### 1.3 The Role of Pain

Autonomic nervous system reactivity is associated with pain sensitivity and tolerance. A meta‐analysis has demonstrated an increase in sympathetic‐baroreflex activity and a decrease in vagal‐parasympathetic activity in response to experimentally induced pain in healthy individuals [24]. Furthermore, meta‐analytic evidence points to decreased HRV in states of chronic pain [25]. Some studies examining the association between pain and HRV in NNSI have found a somewhat different picture. For example, reduced pain sensitivity was found in adolescents with NSSI, which was associated with blunted autonomic and endocrinological responses to experimentally induced pain with respect to healthy controls [26]. Such a blunted response fits well with the results of a neuroimaging study where the authors observed decreased amygdala reactivity after incision (compared to sham) in patients with BPD [27]. Considering that both cross‐sectional and longitudinal studies found an inverse association between changes in HRV over time and symptoms of BPD in adolescents with NSSI [28, 29], we hypothesise that the urge to engage in NSSI would be characterised by reduced HRV. Given that NSSI reduces amygdala activation, regulating intense physiological arousal, we further hypothesise that HRV would increase after engaging in such behaviour.

Therefore, examining any potential correlation between an objective measure of the physiological effects of pain in the context of NSSI (HRV) and subjective distress would be useful. However, the practical implications of diary data collection at times of distress require further understanding.

### 1.4 Affect Regulation With Electrical Vagal Nerve Stimulation (VNS)

It is hypothesised both that NSSI may occur at times of low vagal tone and HRV and that improvements in HRV may occur after NSSI [17]. It is possible that the improved affect regulation often described after NSSI may be associated with and even mediated by increased vagal tone. The possibility that vagal tone mediates improved emotion regulation is supported by a case of improved emotion regulation in clients with autism treated with VNS for epilepsy [30]. Given that there is some evidence that electrical stimulation of the vague nerve is associated with affect regulation, it is reasonable to consider whether mechanical stimulation of vagal pathways plays a role in affect regulation in the context of NSSI, but no attempts to collect HRV data in vivo in NSSI have been made. and the practical implications of this are not understood.

### 1.5 Monitoring of Affective States and Physiology During NSSI

There are many studies that have tried to replicate factors that might cause distress in a laboratory setting. In people with BPD, pictures, films, music, computerised algorithms and personalised scripts have been used [31]. For example, in a study of emotion induction in people with BPD, 96 images from the International Affective Picture System were chosen on the basis that they would present BPD‐relevant stimuli. However, the authors concede that the interpersonal nature of distress is difficult to replicate with pictures [32]. This problem is avoided in an ambulatory setting, where the interpersonal issues that cause distress will inevitably be encountered.

Little is known about monitoring affective states during or immediately prior to self‐harm, although this is becoming technically easier because of smartphone apps and has recently been demonstrated [33]. A recent scoping review has identified that some NSSI intervention mobile apps could be useful in reducing urges to self‐harm [34]. Technology is also enabling physiological measurements, but these objective measures may be perceived as more exposing to the individual with a history of NSSI.

Although the acceptability of long‐term monitoring of distress and HRV in vivo in a population who engage in NSSI is not known, we hypothesise that it is possible. This study aims to test this hypothesis.

## 2 Methods

### 2.1 Study Design

This prospective observational study was designed to examine the feasibility of monitoring HRV during distress and NSSI in real life rather than in a laboratory setting. The participants were students. The strengthening the reporting of observational studies in epidemiology (STROBE) statement: Guidelines for reporting observational studies have been utilised.

### 2.2 Recruitment

The setting was Falmouth University, a specialist university for the creative industries based in Cornwall in the United Kingdom. NSSI is an important issue for the university, which facilitated recruitment to the study. In line with our objective of understanding the feasibility of monitoring HRV during NSSI or distress, understanding the feasibility of recruitment to such studies was an important step. For this reason, we did not have rigid inclusion criteria, and the only such criterion was being a student at Falmouth University. It was decided to recruit 10 students based on discussions between researchers and university staff regarding what would be an achievable recruitment target. Previous or current NSSI was not required for inclusion. However, it was felt to be desirable to include some participants with active or previous NSSI, which would allow some examination of feasibility in this particular group. For pragmatic reasons, the exact proportion of such participants was not stipulated, but this point was made to potential participants at briefing meetings. It was anticipated that distress would be experienced to some extent by some or all of the participants, so the feasibility of monitoring during times of distress would be possible even in the absence of NSSI. Student support at Falmouth University identified student mentors as a suitable group to invite to meetings. Student mentors are senior students who volunteer to mentor other more junior students. They were chosen because this role often involves a pastoral element and an interest in mental health. Many student mentors have experienced mental health issues themselves, but they are less likely than other students to be in a crisis.

Exclusion criteria included self‐disclosed serious current mental illness (bipolar disorder or schizophrenia), alcohol or drug abuse, sociality, or mental health crisis. Self‐disclosure on a pre‐study questionnaire was chosen for pragmatic reasons. There were opportunities to discuss exclusion criteria privately at briefing meetings. Self‐report was considered likely to be accurate in a group of educated people where mental health issues were relatively less stigmatised. Participants were informed that they would be compensated with a £50 Amazon voucher after the study.

Written informed consent was obtained from all subjects.

For the duration of the study, a psychiatrist was available by mobile phone for support and signposting should it be required in case of suicidal feelings or concerns about safety. Contact details of three psychiatrists were available on the app and as part of the information pack.

### 2.3 HRV Monitor

The Bodyguard 2 ambulatory HRV monitor (Firstbeat) is a long‐term ECG‐based recorder with two disposable electrodes that provides standard RR intervals with precision in milliseconds. Both the beat‐to‐beat detection accuracy and artefact correction algorithm of the Bodyguard 2 device have been evaluated in validation studies [35]. This device allows up to a week of monitoring on a single charge. It was agreed that a week would be long enough to allow a reasonable chance of capturing an episode of NSSI.

Bodyguard 2 monitors have been extensively used in HRV research, but not in NSSI [36]. One electrode is placed on the right side of the chest below the clavicle, and the other is placed on the left side of the chest roughly around the position of the cardiac apex. The participants were given pictures showing correct electrode positioning, and the first application of electrodes was checked by researchers. Ambu Bluesensor electrodes were used (as recommended by Firstbeat). Tiga‐med ECG electrodes were provided as an alternative.

### 2.4 Software

Kubios software is well validated for the calculation of the root mean square of successive differences of inter‐beat intervals (RMSSD) [37]. RMSSD is recommended for short‐term components of HRV measurement and is an indicator of vagal tone [38]. Moreover, RMSSD is less affected by breathing and is therefore recommended for field studies [39, 40].

Artefacts were corrected using the Kubios automatic artefact correction algorithm. This software uses an advanced detrending method based on smoothness prior formulation in which the filtering effect is attenuated in the beginning and the end of the data, thus avoiding the distortion of data end points. Furthermore, the frequency response of the method is adjusted with a single smoothing parameter, selected in such a way that the spectral components of interest are not significantly affected by the detrending. Kubios HRV includes two methods for correcting any artefacts and ectopic beats: (1) threshold‐based correction, in which these are simply corrected by comparing every RR interval value against a local average interval; and (2) automatic correction, in which artefacts are detected from a time series consisting of differences between successive RR intervals.

### 2.5 Diary App

A prototype diary app (for the android platform) was designed by researchers at Falmouth University. An app‐based system of recording NSSI, distress and diary entries was chosen because it is more convenient than a paper based system. It also allows the precise date and time of entries to be recorded. Furthermore, it provided a convenient means of providing safety information and contact numbers to participants. Participants were asked to record a routine check‐in with diary data and subjective units of distress score (SUDS) [41]. This was done twice a day (in the morning and evening) and also at times when they felt distressed. Diary data were stored on the phone and downloaded to a secure laptop after the end of the week.

### 2.6 SUDS Scores

It was decided to use SUDS rather than more general measures of emotion for simplicity. On a SUDS scale, zero represents an absence of distress and 10 the highest level of distress possible [41]. It was anticipated that instances where the urge to self‐harm was high would correlate with high subjective levels of distress.

The app (Figure 1)

- prompted the entry of free text information about feelings
- recorded a SUDS
- offered contact numbers and signposting in case of serious self‐harm requiring urgent intervention.

**FIGURE 1:**
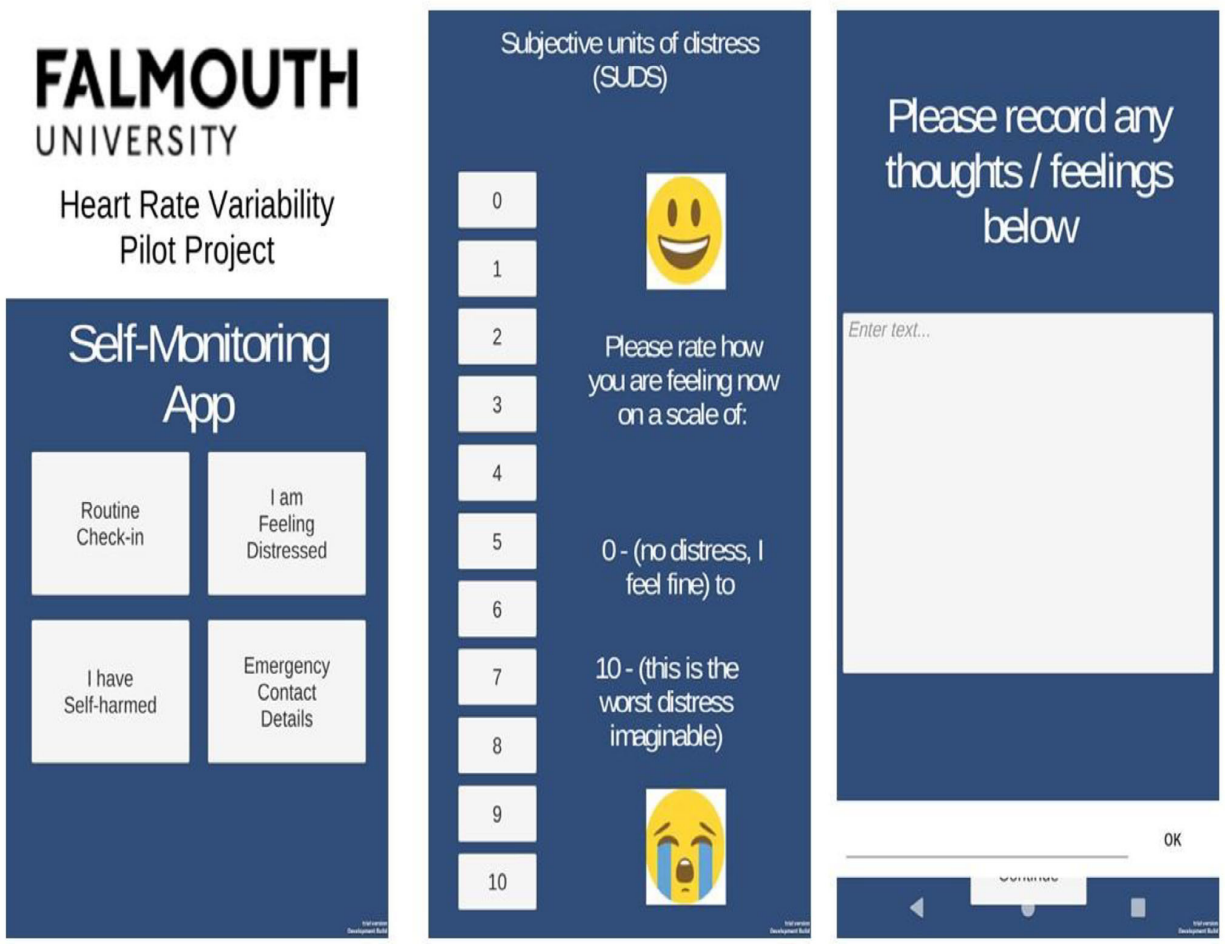
A screenshot similar to one from the app for illustrative purposes.

### 2.7 Questionnaire

A questionnaire was used to assess the participants’ experience (including physical and emotional effects) of wearing the HRV monitor for a week. It was designed to establish whether participation was safe and convenient. It used largely open questions to allow a wide range of possible responses.

### 2.8 Ethics

The study was approved by the ethics committee of Falmouth University (ref: RIEC 1906 14/02/2019). Written informed consent was obtained from all participants. No participant can be identified from the presented data.

### 2.9 Patient and Public Involvement

Development of the project was discussed and supported by experts by experience at various stages. At the start of the study, the project team engaged with student welfare mentors at Falmouth University to discuss the proposed plan. Two open engagement events of two hours each were advertised across the university with an open invitation to anyone interested in mental health issues. The events were led by the student welfare mentors and the project team to capture priorities, experience, and preferences. Forty young people in total attended the two events, including a mix of people who had self‐harmed, had mental health issues, or those with no past history but an interest in the topic of mental health. The events explored the mental health issues the attending group had experienced, the potential of technology to enable them to understand and monitor their feelings and responses, and the type of technology that may be used in the project. The concept of using a digital diary to capture participant experience emerged in the first event. All 10 of the study participants who later volunteered to be in the study had been involved in the initial study design events. The study results are to be fed back to all participants after publication.

## 3 Results

Ten students were recruited to the study. Participants’ characteristics are provided in Table 1.

**TABLE 1:** The characteristics of participants.

Average age	20.6 years (range 19–22)
Male: female	3:7
History of NSSI (ever)	6 (60%)
Current NSSI (within a year)	2 (20%)
Current psychotropic medication	1 (10%)

The types of NSSI which the participants had used (number of individuals in brackets) included cutting [3], burning [3], hitting [2], scratching, overdosing, strangulation, biting, pinching, and holding objects with a lot of pressure. No participants disclosed a history of eczema, but three had a history of allergy/asthma. During the study, no participants engaged in NSSI or had the urge to do so.

The subjective experience of the participants was assessed in the post‐study questionnaire with the question: ‘Please describe your overall experience of taking part in the study.’ There were also questions on the app and the monitor. Table 2 shows participant comments about their overall experience.

**TABLE 2:** Overall participant experience and other comments.

Participant	Response
A Current NSSI	*Overall experience*: As normal life. Quickly grew used to the heart monitor and no one noticed it unless I mentioned it. Slight itching if electrodes not replaced often enough‐ only a minor discomfort though. App suggestion‐ may be interesting for the app to be able to record excessive positive emotions. Study suggestion‐ longer length of time to capture emotional states.
B Previous NSSI	*Overall experience*: Overall experience was extremely positive. I had no issue wearing the heartbeat monitor when going about my day to day life. It was a great feeling to be helping in this study. Slight skin irritation App‐ it was extremely effective and simple to use, but there should be a version for other devices like iPhones.
C NSSI never	*Overall experience*: It was an interesting experience, I wouldn't say I incurred anything negative. Working with the app and monitoring my feelings made me pay more attention to them and eventually helped me deal with them. My skin got very irritated and it became painful to change them (the electrodes). App suggestion‐ it was very easy to use. Maybe it would be great to have confirmation after submitting an entry. Participating led to some interesting conversations, no especially strong emotions but conversations about self‐harm and mental health, which made me feel uncomfortable at times.
D Previous NSSI	*Overall experience*: I had no problems with wearing the monitor other than the skin irritation. People did ask but I found it easy to communicate what it was for. The experience was fine. Skin irritation People did ask (about the monitor) but I found it easy to communicate what it was for App—it would have been useful to give the user confirmation that it has been logged. Irritating that when the phone fell asleep if I sat and thought everything I wrote disappeared.
E NSSI never	*Overall experience*: It was good, can't really feel that I'm wearing the monitor. However, I feel irritated/ itchy where the sticky bits are placed towards the end of the week. (The skin around that area are red & painful). Irritated/ itchy where the sticky bits are placed towards the end of the week. A little bit uncomfortable when lying on left side. App‐ too long to load up, interface could be improved, notifications/reminders would be useful, being able to look at previous entries would be nice. Emotions taking part in the study “Not sure, I felt like I get irritated easier but it could be from lack of sleep.”
F Previous NSSI	*Overall experience*: My experience taking part in this study was fine. I didn't treat my day with the HRV monitor any different from my normal activity. I did get a bit of an irritation from one on the batch of electrodes. App‐ wasn't really a clear scale to express how one was feeling. Often forgot…some form of a notification system as a reminder would help. Did have a night terror on the first night.
G Previous NSSI	*Overall experience*: Positive. It didn't have much of an impact/ restrict my day to day life. The app and monitor were easy to use and instructions/ briefings were well communicated. Sticky pads did irritate skin a little bit but it was a minor thing. The pads lost their stickiness. App—[having to carry a second phone with the app] did mean I occasionally forgot to have it with me. A bit more instruction on how to document thoughts/ feelings, and a bit more of a guideline for how to score distress.
H Current NSSI	*Overall experience*: It was easy to take part in and the only extra stuff that add into my day to day routine was making a short routine check in 2 times a day and altering the heart rate monitor. The only discomfort I had came with the pads, they came itchy after a while…irritated my skin. App—making it more clear what it meant by “distressed”. Somewhere separate for alcohol intake.
I NSSI never	*Overall experience*: It was an overall good experience, no major issues. The monitor is light and didn't bother me wearing it. Experienced a little irritation on the skin but nothing major. Would do it again if I had to. A little irritation on the skin. App—slightly confusing being asked about distress at daily check‐in. The monitor does have a “hospital feeling” that could trigger some people.
J NSSI never	*Overall experience*: Rather smooth, didn't require too much effort or cause a lot of discomfort. Skin got a little irritated. App—fairly straightforward and easy to log experiences. Maybe the app could transfer information without needing to plug the phone in.

In summary, Table 2 demonstrates a generally positive experience, although skin irritation was mentioned by all participants.

Kubios software allows analysis of time spent in each of 5 RMSSD zones, which represent a range from very low vagal tone to high vagal tone. The lowest 2 zones, with RMSSD less than 5 ms and RMSSD between 5 and 12 ms, are periods of lowest vagal tone, which are of particular interest.

Table 3 shows that 9 of the 10 participants were able to use the diary app successfully and all managed to record HRV values for a significant time (between 67 and 176 h).

**TABLE 3:** App and HRV monitoring by participant.

ID	NSSI history	App diary entries and SUDS scores (0‐10)	HRV monitoring		
A	Current NSSI Less than monthly: pinching, biting, holding blunt objects with a lot of pressure, strangulation (light)	27 diary entries SUDS scores: 0,0,0,1,0,2,0,0, 0,0,0,0,0,0,0,0, 1,0,0,0,0,3,0,0, 0,3,0,0,0,0,3	150 h, average RMSSD: 66 ms		
			**Vagal tone**	**RMSSD (ms)**	**Time**
			Very low	<5	2 h
			Low	5–12	8 h
			Lowered	12–27	28h
			Normal	27–72	82 h
			High	>72	31 h
B	Previous NSSI 5 or 6 years ago: cutting	10 diary entries SUDS scores: 0,0,2,2,3,0,0,0,1,0	67 h, average RMSSD: 90 ms		
			**Vagal tone**	**RMSSD(ms)**	**Time**
			Very low	<5	0 h
			Low	5–12	1 h
			Lowered	12–27	8 h
			Normal	27–72	26 h
			High	>72	31 h
C	NSSI never	No diary entries or SUDS scores due to technical fault	127 h, average RMSSD: 31 ms		
			**Vagal tone**	**RMSSD (ms)**	**Time**
			Very low	<5	2 h
			Low	5–12	4 h
			Lowered	12–27	43 h
			Normal	27–72	77 h
			High	>72	1 h
D	Previous NSSI 9 years ago: burning with hot spoon, hitting self	15 diary entries SUDS scores: 3,2,0,2,4,1,3,3,2,0,5,1,2,0,5	176 h, average RMSSD: 28 ms		
			**Vagal tone**	**RMSSD (ms)**	**Time**
			Very low	<5	3 h
			Low	5–12	23 h
			Lowered	12–27	99 h
			Normal	27–72	50 h
			High	>72	2 h
E	NSSI never	11 diary entries SUDS scores: 1,5,3,3,2,1,3,3,5,4,3	145 h, average RMSSD: 104 ms		
			**Vagal tone**	**RMSSD (ms)**	**Time**
			Very low	<5	2 h
			Low	5–12	3 h
			Lowered	12–27	6 h
			Normal	27–72	42 h
			High	>72	92 h
F	Previous NSSI last year: overdose Burn	9 diary entries SUDS scores: 6,0,0,0,0,0,0,0,1	161 h, average RMSSD: 38 ms		
			**Vagal tone**	**RMSSD (ms)**	**Time**
			Very low	<5	2 h
			Low	5–12	5 h
			Lowered	12–27	51 h
			Normal	27–72	98 h
			High	>72	4 h
G	Previous NSSI 2 years ago: cutting, punching surfaces	19 diary entries SUDS scores: 2,0,5,7, 2,8,7,0,3,6,1,1, 0,3,1,1,6,6,5	160 h, average RMSSD: 85 ms		
			**Vagal tone**	**RMSSD (ms)**	**Time**
			Very low	<5	3 h
			Low	5–12	2 h
			Lowered	12–27	20 h
			Normal	27–72	52 h
			High	>72	83 h
H	Current NSSI: scratching Previous NSSI: cutting, burning	19 diary entries SUDS scores: 3,1,1,3, 2,4,0,2,1,4,3,1, 0,2,1,2,1,3,6	168 h, average RMSSD: 47 ms		
			**Vagal tone**	**RMSSD (ms)**	**Time**
			Very low	<5	4 h
			Low	5–12	11 h
			Lowered	12–27	33 h
			Normal	27–72	94 h
			High	>72	26 h
I	NSSI never	9 diary entries SUDS scores: 1,3,1,5, 0,0,0,0,1	92 h, average RMSSD: 118 ms		
			**Vagal tone**	**RMSSD (ms)**	**Time**
			Very low	<5	4 h
			Low	5–12	3 h
			Lowered	12–27	5 h
			Normal	27–72	9 h
			High	>72	72 h
J	NSSI never	8 entries SUDS scores: 2,9,6,2, 3,3,6,4	121 h, average RMSSD: 62 ms		
			**Vagal tone**	**RMSSD (ms)**	**Time**
			Very low	<5	2 h
			Low	5–12	6 h
			Lowered	12–27	9 h
			Normal	27–72	62 h
			High	>72	43 h

### 3.1 Diary App

The app was used successfully by nine out of 10 participants. Due to a software problem, diary entries were not saved in one case, although that participant did use the app. This may have been due to slight differences in the operating system between different Android phones. An average of 14 diary entries was made.

Three participants commented on the lack of a notification system and that they sometimes forgot to make diary entries. Three participants commented that they were unsure how to interpret the SUDS scale. Two participants said that the lack of confirmation that an entry had been recorded was problematic and that the app timed out, losing whatever data had been entered.

### 3.2 HR Monitor

HR recordings were done for an average of 136.6 h (range 66.9 to 176.3). All participants wore the device on most or all of the seven days. Gaps in recording occurred due to discomfort sleeping with the monitor or due to skin irritation as well as for washing.

Ten out of the 10 participants reported that some skin irritation developed after a few days of wearing the electrodes. One participant tried moving the electrodes slightly each day and commented that there was no suitable skin left for electrode attachment by the end of the week. Both brands of electrodes used caused skin irritation.

Wearing the monitor resulted in an ‘uncomfortable conversation’ about mental health in one participant, and another commented that the monitor had ‘a hospital feeling, which could trigger some people.’

None of the participants made contact with the psychiatrists for emotional support during the study.

### 3.3 Linking HRV to NSSI

It was not possible to analyse HRV at times of NSSI or the urge to self‐harm during this project because no such instances occurred. One participant commented that a longer period of monitoring would be required to capture episodes of self‐harm.

### 3.4 Linking HRV to Subjective Distress

Due to incorrect synching of date and time on the HRV monitor in some participants, it was only possible to accurately link diary entries and SUDS scores with measures of HRV (RMSSD) in 4 cases. Using Spearman's coefficient, no statistically significant correlation between RMSSD and SUDS score was found in these 4 cases.

## 4 Discussion

Monitoring ambulatory HR for up to a week using chest electrodes and a heart rate monitor was possible in a group of students, 6 of whom have a history of NSSI at any time and 2 of whom were currently engaged in episodes of NSSI (as defined by NSSI within a year). The use of a diary app is acceptable and practical, although improvements should be made. Further work is required to calculate the duration of monitoring and/or the number of participants required to test the hypothesis that NSSI occurs at relatively low HRV for that individual, and that HRV subsequently improves.

This study suggests that skin irritation would be a barrier to extended periods of monitoring using chest electrodes. Therefore, in order to capture HRV data during self‐harm, a future study would require careful selection of a subset of people who self‐harm frequently, which may make recruitment of significant numbers challenging. Alternatively, as HRV monitoring technology rapidly improves, a less invasive way of collecting accurate inter‐beat intervals may become possible. A chest strap has been shown to accurately record HRV and does not require adhesive electrodes [42]. However, this method would require high levels of cooperation from the participant and can be uncomfortable if used for long periods. Devices worn on the wrist are becoming more accurate and could be considered for future research, but accuracy is affected by emotional stress [43]. It may be that slightly less accurate data would be an acceptable trade‐off for a non‐invasive method of HRV recording that would allow monitoring over a period of months.

No major adverse psychological effects of participation were found. Minor issues included one uncomfortable conversation about mental health, irritability in one participant, and a comment about a ‘hospital feeling’ being a potential trigger for some. This may reflect the on‐going stigma associated with mental health difficulties. The large number of positive comments made by participants bodes well for further research in this area.

The app requires improvement if it is to be used in a larger‐scale diary monitoring exercise. Testing it on the slightly different operating systems to ensure reliable recording and retrieval of data would be useful to avoid loss of data. Participants found the SUDS scale difficult to use, so an alternative method of describing affect, like the positive and negative affect scale (PANAS), which has been extensively used in psychophysiological research, should be considered in future studies [44]. A notification system to prompt, confirm, and amend diary entries would be useful to prevent instances where participants forgot to enter data or where it was entered but not saved.

In future designs, consideration should be given to correcting for HRV effects of activity (e.g., by incorporating an accelerometer). In addition, HRV measurements taken at the time of diary entries should be made at rest and in the same body position each time to allow for better comparison between HRV measures.

No statistically significant correlation between SUDS distress scores and HRV was found in the 4 participants where this was measured. A robust interpretation of data in this number is not possible, but it is in keeping with the results of a 24 h ambulatory monitoring study with subjective measures of distress where there was no interaction effect between distress and HRV, which the authors found surprising [45]. These findings may be easier to understand if emotion regulation capacity is thought of as a self‐regulatory ‘muscle’ [46, 47]. At times of stress, with regulatory effort, HRV may even increase, but if overwhelmed, HRV can then drop like strength in a fatigued muscle with consequent failure of top‐down control.

In future projects, if using the same HR monitoring method as in this study, it would be important to ensure that date and time information between app and monitor were properly synchronised on all devices to ensure that valid comparisons between the app data and HRV data can be made.

No instances of NSSI occurred during this project. It was not possible to test the hypothesis that NSSI occurs at times of low HRV when top‐down emotional regulation capacity is overwhelmed. It was also not possible to do a power calculation to establish the number of participants required or the duration of monitoring required to test the hypothesis.

The involvement of people with lived experience of NSSI in the design of the study was a strength. However, in future studies, in order to ensure robust sample selection, it would be better for those involved in any aspect of study design not to be included as participants.

The main limitation of such a small test group would be the impact on the internal and external validity of the study. However, as this was a feasibility study it provided invaluable insights into the practical challenges that need consideration.

### 4.1 Implications and Recommendations

This study provides evidence that chest‐based ambulatory HRV monitoring in people with a history of NSSI is acceptable for a period of up to a week, but the duration of ECG monitoring is limited due to skin irritation. A longer duration of monitoring would allow a higher probability that NSSI episodes would occur during the monitoring period. As wrist‐based technologies improve, this may be an alternative for a longer duration of ambulatory monitoring.

The study findings also suggest we reflect on whether all possible opportunities to use wearables were investigated. This includes considering technology such as rings and smartwatches which could reflect HRV or be modified to do so. In future work it should be imperative that prior to embarking on a feasibility study, all available technology is presented to experts by experience and clinicians to discuss and identify which might be best suited for the feasibility trial.

Improvements to the app are required to increase its practicality for research. It is recommended that the app should send reminder notifications and capture and save input as it is entered to prevent lost data due to timeouts. Using SUDS as a measure of distress was confusing to some participants. Alternative subjective measures of affect (like the PANAS) should be considered. It would be useful for participants to practice data entry during an induction process so that they are familiar with the measure of affect used and data entry on the app. Careful synchronising of devices (between the diary app and HRV monitor) is necessary when collecting time‐sensitive data using two methods to allow accurate correlation between subjective diary entries and HRV data.

The nature of the relationship between HRV (as a measure of vagal tone), distress, and the strength and activity of top‐down self‐regulatory effort is still unclear. The possibility that the relationship is non‐linear should be further explored. It is possible that NSSI occurs at times of low vagal tone and that vagal tone subsequently improves. NSSI may increase vagal tone [17]. Examining these questions using ambulatory monitoring and a diary app is feasible in clients with a history of NSSI.

Another important approach that needs consideration alongside our proposed approach is the more recently postulated digital phenotyping [48]. This approach could allow the collection of real‐time data such as physiological responses, mood and sleep. Coupling this passive data collection with the active data insights via the Diary app and HRV monitoring could allow for triangulation and development of prediction models of times of ‘high risk’ during the day for NSSI [49].

## Author Contributions

**Alje van Hoorn**: conceptualisation, data curation, formal analysis, investigation, methodology, validation, visualisation, writing – original draft. **Anna Mankee‐Williams**: investigation, methodology, project administration, resources, visualisation, writing – review and editing. **Gareth Lewis**: investigation, project administration, resources, software, validation, visualisation, writing – review and editing. **Rafaella Mellili**: investigation, methodology, visualization, writing – review & editing. **Jessica Eccles**: investigation, methodology, visualisation, writing – review and editing. **Cristina Ottaviani**: investigation, methodology, visualisation, writing – review and editing. **Richard Laugharne**: investigation, methodology, visualisation, writing – review and editing. **Rohit Shankar**: conceptualisation, data curation, formal analysis, funding acquisition, investigation, methodology, project administration, resources, supervision, validation, visualisation, writing – review and editing.

## Conflicts of Interest

Rohit Shankar has received institutional and research support from LivaNova, UCB, Eisai, Veriton Pharma, Neuraxpharm, Bial, Angelini, UnEEG and Jazz/GW pharma outside the submitted work. No other author has any declared conflict of interest related to this paper.

## Data Availability

All data used for the paper is within the manuscript.
